# Regulating pharmaceutical companies’ financial largesse

**DOI:** 10.1186/s13584-018-0220-5

**Published:** 2018-05-14

**Authors:** Ronen Avraham, Charles Silver

**Affiliations:** 1 The Buchmann Faculty of Law, Tel Aviv University, Tel Aviv, Israel; 2 University of Texas, Austin, USA

## Abstract

Nissanholtz-Gannot and Yenkellevich (NGY) explore the impact of a 2010 amendment to the Israeli National Health Insurance Law that requires annual reporting of payments from pharmaceutical companies (PCs) to doctors and healthcare organizations. The amendment was adopted to ensure transparency and to facilitate appropriate regulation of interest conflicts. To learn whether the amendment was having the desired effects, NGY interviewed multiple representatives of an assortment of stakeholders. They found broad agreement among the respondents that financial relationships between PCs and physicians should be transparent. But they also discovered that ignorance of the 2010 amendment was widespread, especially among physicians, and that knowledgeable respondents thought loopholes rendered the law ineffective. Lastly, NGY found that the improvement in the transparency culture has more to do with pressure put by international and non-Israeli national actors on the multi-national PCs operating in Israel than with the Israeli new law.

In this short paper we critically review NGY’s study. We are much less optimistic than they are about the situation in Israel. For example, we show that the new law has not increased transparency vis-à-vis the patients as virtually all reports to the government specify only the institutions receiving them and not individual physicians’ names. We are skeptical of the effectiveness of self-regulation or government regulation. Instead, we propose some ways to increase patients’ oversight, such as facilitation of class actions to enforce fiduciary duties and disclosures, as well as structuring co-payments for drugs in ways which will signal to the patients their relative efficacy.

## Background

Nissanholtz-Gannot and Yenkellevich (NGY) explore the impact of a 2010 amendment to the Israeli National Health Insurance Law that requires annual reporting of payments from pharmaceutical companies (PCs) to doctors and healthcare organizations when the value conferred exceeds 2500 NIS. The amendment was adopted to ensure transparency and to facilitate appropriate regulation of interest conflicts. According to reports filed with the Israeli Ministry of Health (MOH), tens of millions of dollars are transferred by PCs every year [1].

To learn whether the amendment was having the desired effects, NGY interviewed multiple representatives of an assortment of stakeholders. They found broad agreement among the respondents that financial relationships between PCs and physicians should be transparent, meaning that payments from the former to the latter should be disclosed. But they also discovered that ignorance of the 2010 amendment was widespread, especially among physicians, and that knowledgeable respondents thought loopholes rendered the law ineffective. For example, the law allows PCs to “channel [] payments to physicians without public scrutiny” by engaging them as consultants, compensating them for lectures, sponsoring organizations that appoint them to their boards, funding post-marketing research, and paying hotel charges and registration fees for doctors who attend conferences abroad. Interviewees implied that many such payments were excessively lavish, their purpose being to buy loyalty rather than to cover actual costs. Many respondents added that lax enforcement by the MOH weakened the law as well.

In contrast, the interviewees had more positive things to say about private regulation, which takes the form of ethics codes adopted by multinational PCs, their umbrella organizations, medical associations, and employers. These codes “[brought] issues of transparency and accountability to the fore,” led many PCs to restrict gifts to doctors, and encouraged the use of contracts that compensate doctors and organizations for providing services of identified types in place of unrestricted gifts. Self-regulation also led PCs to route support payments to physicians via employers, instead of giving directly to physicians as before. In practical effect, the PCs and the health plans that employ Israeli physicians are responsible for ensuring adherence to the ethics codes that the involved business entities and organizations have adopted. To the extent that they succeed, the burden on public enforcers like the MOH is reduced.

Finally, NGY studied official reports and found substantial differences between payments reported to the MOH as having been made and payments reported as having been received. This discovery supports the opinion of the interviewees that enforcement of the disclosure law by the MOH is spotty. It may also reflect ambiguities in the 2010 law that foster disagreements between donors and recipients regarding the money transfers that must be reported.

It is important to recognize that the NGY study does not attempt to evaluate the extent to which Israeli physicians are unduly influenced by PCs. This is literally (actually much more than) a million dollar question, though one which NGY cannot answer. Instead they report an improvement in the perceived ethical environment in Israel. And yet, they report that the perceived improvement has more to do with pressure exerted by international and non-Israeli national actors on the multi-national PCs operating in Israel than by the new Israeli law or any enforcement of it by the MOH [2].

## Commentary

When discussing the importance of their findings, NGY are more sanguine than we think is appropriate. For example, they see the shift from gifts to contracts as a fundamental change that makes corruption less likely. “Contractual relations imply a different logic than gift exchanges,” they write, adding that the former “do not foster any relationship or sense of obligation beyond the terms of the contract.” We disagree. In a competitive market, one may expect a contract to require the payment of an appropriate price, but the “market” in which PCs give money to doctors and health care organizations may not fit this description. PCs may not be shoppers looking for good deals, and physicians-recipients may not be suppliers competing for sales. NGY neither examined any contracts nor assessed the reasonableness of the agreed-upon prices. For all anyone seems to know, the contracts they applaud could just be formalized means of conferring lavish bribes. Moreover, even in competitive markets, sellers hoping to earn repeat business work hard to keep their customers happy. Doctors and health care organizations may seek to curry favor with PCs for the same reason. They may want the stream of payments to continue to flow.

The effectiveness of private regulation of interest conflicts can also be debated, as NGY are aware. For example, the code of conduct established in 2002 by the Pharmaceutical Research and Manufacturers of America allows PC representatives to provide meals and educational gifts. [3] Restricting these emoluments has been shown to alter the prescribing practices of physicians who work at academic medical centers [4].

Even small gifts, then, can cause physicians to depart from the ethical requirement to “act for the good of the patient” rather than for the good of a donor [5]. This is merely one aspect of a more general problem: No incentive structure can fully align the interests of agents (here, health organizations and physicians) perfectly with those of the principals they serve (here, patients). For example, in the United States patients may find it convenient to visit doctors who provide imaging services, infusions, blood tests, radiation treatments, physical therapy, or other services in-house, but the prospect of profiting off these ancillary services may cause doctors in the U.S. to over-prescribe them. As a result, needs arise for monitoring and other supplements that ensure agents’ loyalty. Legal duties, patient monitoring, regulation, and competition all exert some amount of pressure on providers to ignore temptations to put their interests ahead of those of their patients.

Israel does not face these exact problems as physicians rarely have financial stakes in these sorts of ancillary services. Yet, Israel has developed a variety of means to deal with physicians’ pharma-related interest conflicts. It uses a centralized governmental process to determine whether new drugs, which tend to be expensive, will be covered by the basic benefits package that the country’s non-profit health plans must offer. The health plans typically do not cover drugs that are rejected at the national level, though there have been some notable exceptions to this, as in the case of the coverage of certain cancer drugs by Maccabi (one of Israel’s HMOS) [6]. Moreover, physicians can ask their health plans for special authorizations of coverage for specific cases, and sometimes these requests are approved. In the absence of generic coverage of a drug or case-specific authorization, a physician can still prescribe what she believes is effective, but the health plan will not cover the cost and this is communicated to the patient on the prescription itself. In light of all this, in Israel there are significant limitations on the physician’s’ ability to secure medications not authorized by patients’ health plans, even if induced to do so by favors or indoctrination from pharmaceutical companies.

At the same time, it should be noted that physicians are required to alert their patients to the existence of the most effective medications for their conditions, even if these are not in the national or health plan formularies. In addition, all health plans have “exceptions committees” which consider requests from patients to cover medications for them on an individual exceptions basis [7].

In stark contrast to the United States, where doctors have unlimited discretion to prescribe medications, Israeli physicians who work for health plans generally must adhere to guidelines set by their employers. The health plans restrict prescriptions of drugs not in the approved formulary, suggest alternatives to prescribed drugs that cost less, educate doctors about drug costs, and discipline doctors whose prescribing habits are unusually expensive [8].

Given these constraints, concerns about interest conflicts with the potential to harm patients should be directed more at health plans, hospitals, and other organizations that employ doctors and less at physicians themselves. Given their managerial powers, these organizations are the natural targets of PCs’ largesse. Why attempt to bribe physicians when their freedom to promote the use of pricey medications is so greatly constrained?

In keeping with the point just made, the 2016 reports issued by Israel’s MOH do not show a single donation to a physician from a PC. Instead, tens of millions of dollars were transferred from PCs directly to HMOs, hospitals, NGOs, medical schools, research institutes, and the like. That said, a substantial portion of this money was nonetheless reportedly used to cover charges associated with conferences abroad, hotels, airfare, and other items that benefit physicians [9]. While the reports make transparent the monies that PCs gave, they do not reveal the identity of the doctors who received them. If donating directly to physicians had been illegal, then, the reports would have revealed something in the nature of organized crime, where dollars flow to bosses who distribute it to anonymous underlings. Seen from this angle, control of the flow of dollars from PCs to physicians enhances the ability of HMOs, hospitals and other employers to influence and regulate physicians’ prescribing practices. Whether these organizations use their enhanced powers to cause doctors to serve patients better or less well is the question of primary interest. NGY’s interviews shed no light on this subject, but the strong performance of Israel’s health system gives one reason to be optimistic.

And yet in 2010 the Israeli legislature thought public regulation of PCs and physicians’ behavior was desirable. However, if it is conceded that a need for public regulation exists, then NGY’s article is most useful for the lessons it teaches about regulatory design. Judging from the views expressed by NGY’s interviewees, Israel’s reporting regime is ineffective because it was adopted without industry buy-in, is poorly enforced, and is also easily circumvented. NGY suggest fixes for some of these problems, especially closing loopholes that exempt many kinds of wealth transfers from the reporting requirement and strengthening enforcement by the government.

Unfortunately, enacting desirable conflict regulations is difficult in democracies, where interest groups exert considerable influence on lawmaking processes. NGY do not say whether interest groups are responsible for the shoddy design of Israel’s disclosure law, but they nicely document the regime’s myriad flaws: loopholes that exempt compensation that doctors receive directly or indirectly from PCs for consulting, delivering lectures, serving on boards of patient advocacy groups, conducting meaningless post-marketing studies of the effectiveness of drugs, and attending conferences at fancy hotels; a definition of the term “payment” that exempts business expenses that benefit physicians, such as reducing registration fees for conferences by funding organizers; and the complete failure of the MOH to enforce the disclosure regime’s prohibitions. In view of these serious deficiencies, it should surprise no one to learn that the law is widely ignored. Eighteen of the 46 stakeholders they interviewed “were not aware of the existence of the law requiring disclosure of payments by PCs, and none of the remaining interviewees claimed to be thoroughly familiar with it.”

What can be done then to reduce interest conflicts? As NGY show, government regulation is often partial and toothless, and even the celebrated self-regulation by the industry may be, at least in part, a pretty façade that conceals a less pleasant reality behind it. NGY propose to tighten the regulation by closing loopholes. Unfortunately, government regulation is rarely a silver bullet. Interest group politics, the revolving door that connects industry to government, deficient enforcement incentives, and resource limitations can frustrate good intentions. Market actors subject to regulation are too often at least one step ahead of the regulator in their quest to circumvent the regulation.

Under most disclosure regimes, doctors report their financial interests to a government agency or some other entity. The information they report is then logged and sometimes published online. This is the situation in Israel. Two obvious limitations with this approach are that patients rarely check their doctors’ reports and lack the sophistication needed to evaluate the importance of the information and to understand its implications. In Israel, they cannot even in theory check the names of their physicians online, because those are hidden behind the “corporate shield”- only organizations appear under the online list of recipients. A more desirable arrangement would recognize these problems and address them.

An option that focuses on patients’ legal rights could be desirable, alone or in combination with Israel’s existing disclosure regime. In many jurisdictions in the U.S., the law subjects doctors to both a duty of care to their patients and a fiduciary duty to put their patients’ interests ahead of their own. Israel is no different. For example, Section 7 to the 2014 joint ethical code of the Israeli Medical Association and the representatives of PCs operating in Israel states very clearly that doctors’ first commitment is to their patients, that they should avoid conflicts of interest, and that they should be transparent about conflicts that happen to arise [10].

The fiduciary duty requires doctors to act in good faith, to be loyal, to avoid interest conflicts, and to deal with patients fairly. Physicians who are subject to the duty must disclose any and all financial pressures to which they are subject that could color their treatment recommendations to patients. The duty also requires physicians to communicate openly and fully with patients, so that patients can competently evaluate treatment recommendations and intelligently waive any interest conflicts that are disclosed. Liability for failing to reveal conflicts and for failing to obtain informed conflict waivers is strict. A breach of the fiduciary duty may be found even when a patient is not seriously harmed. As far as we know, Israeli courts have not imposed similar duties on physicians. If they did, Israeli doctors would be legally obligated to directly disclose their financial interest conflicts their patients.

Recognizing patients’ legal rights, courts presiding over medical malpractice cases could require doctors, directly or via their staff employees, to spend more time teaching patients about their legal rights. This could include written or oral disclosures that identify all of the sources from which physicians receive any compensation relevant to a specific patient’s case. In a conversation with a patient diagnosed with an identified malady, a doctor would not only explain the alternative possible treatments and their relative merits and risks, but would also reveal any financial interests that exist other than payment by the patient or the patients’ insurer. In theory, the knowledge that patients who were not told about conflicts might sue could motivate providers to nurture a culture of disclosing. The fear of medical malpractice claims has sometimes has this effect.

Although this approach may have merit, it too faces serious limitations. Because medical malpractice lawsuits are few and far between, disclosure failures are unlikely to be policed. The legal pressure on physicians to make appropriate efforts to educate patients may therefore be insufficient. Moreover, when information about interest conflicts is presented on a stand-alone basis, patients may not know what to make of it. Upon learning that a physician has financial ties to a PC, for example, a patient may not know whether the payment signals a corrupt relationship or is evidence of a physician’s prominence. In one case, a patient should be wary of obtaining treatment from the physician; in the other, a patient should be eager to do so. A patient who learns that a provider generates income by providing ancillary services like scans and blood tests in-house faces a similar conundrum. The income may signal an incentive to deliver services that are unnecessary and potentially harmful or it may indicate a business model that helps patients by conserving their time. In the current climate it might seem unrealistic to expect physicians to emphasize the negatives associated with conflicts, and emphasizing the negatives may not be beneficial for patients either.

We believe there might be a way to overcome these difficulties however. Our goal is to structure the law so that private legal enforcement will be possible. We have little reason to believe the MOH will suddenly start monitoring PCs, or that if it does, it will be able to outsmart them. As the chances that individual patients will file lawsuits against their doctors are slim, one needs to structure the law so that class actions will be possible. Two types of class actions come to mind. First, class actions against HMOs, hospitals and the like for not adequately training their physicians to disclose financial conflict of interest. Second, class actions against PCs for not monitoring the organizations to which they donate so as to ensure that patients learn about possible conflicts of interest. Thus the law needs to explicitly determine that responsibility to inform patients about financial conflicts lies not only on individual doctors but also on their employers, as well as on PCs operating in Israel. Such a regime might not totally cure the system, but it might well improve it significantly.

## Conclusion

We have offered ways to strengthen patients’ oversights of their physicians. Another option is enhancing competition. The best protection consumers of all types can have against interest conflicts is providers’ profit-motivated desire to provide better services at lower cost. In Israel, the United States, and other countries where third-party payers, including government agencies as well as private insurers, control the flow of dollars, competitive pressures are impaired. The possibility of restoring them by delivering more services on a first-party payment basis should therefore be considered. For example, some countries in Europe have a system of tiered copayments where the most effective, or cost-effective, drugs are assigned a lower copayment, while less preferred drugs are assigned higher copayments (Barnieh et al. 2015). If we require doctors to not only reveal the risks and benefits of various alternative drugs but also the size of the copayments associated with them, patients might get reliable signals on the desirability of drugs for them just by looking at the co-payments.
